# Uptake and translocation of polycyclic aromatic hydrocarbons (PAHs) and heavy metals by maize from soil irrigated with wastewater

**DOI:** 10.1038/s41598-017-12437-w

**Published:** 2017-09-22

**Authors:** Shichao Zhang, Hong Yao, Yintao Lu, Xiaohua Yu, Jing Wang, Shaobin Sun, Mingli Liu, Desheng Li, Yi-Fan Li, Dayi Zhang

**Affiliations:** 1 Beijing Key Laboratory of Aqueous Typical Pollutants Control and Water Quality Safeguard, School of Civil Engineering and Architecture, Beijing Jiaotong University, Beijing, 100044 PR China; 2 School of Environment, Tsinghua University, Beijing, 100084 PR China; 3 Lancaster Environment Centre, Lancaster university, Lancaster, LA1 4YQ UK; 4 IJRC-PTS, School of Municipal and Environmental Engineering, Harbin Institute of Technology, Harbin, 150090 China

## Abstract

By investigating the uptake of 16 priority polycyclic aromatic hydrocarbons (PAHs) and five heavy metals from soils to maize at the farmlands with industrial wastewater irrigation, this study revealed the effects of heavy metals on PAHs uptake in terms of co-contamination. The results of 15 investigated soils showed medium contamination level and the vertical PAHs distribution in soils indicated that 2–3 rings PAHs with low octanol-water partition coefficient (log K_ow_ < 4.5) were easier to transport in soils, causing a great potential risk immigrating to the groundwater. The 3-ring PAHs were most likely to be taken up by maize roots whereas 2- and 4–6 ring PAHs had the lower likelihood. The translocation of PAHs in maize tissues has positive relationship with log K_ow_ less than 4.5, while negatively correlated otherwise. Redundancy analysis indicated the unexpected results that, except for soil PAHs concentration, the PAHs translocation by maize was reduced by Pb uptake, but not significantly affected by soil organic matters, pH or the other four heavy metals (Cr, Cu, Ni and Zn). This study for the first time provides the restricted factors of PAHs and heavy metal acropetal translocation by maize when they co-exist at wastewater irrigation sites.

## Introduction

Given the fact of freshwater shortages and economic demands, untreated or partially treated wastewater reuse is widely practiced for agricultural land irrigation, particularly in arid areas at a global scale^1,2^. Using wastewater for irrigation causes the deterioration and the accumulation of contaminants like polycyclic aromatic hydrocarbons (PAHs) and heavy metals in groundwater, which raises concern about public exposure to contaminants in the environment^3,4^. Many previous studies have addressed the sources, accumulation and transfer of PAHs or heavy metals in wastewater-irrigated soils^3,5–7^, but not characterizing the bioaccumulation in case of their co-existence and whether it can affect their distribution in food chain. Wastewater treatment plant is widely recognized as an important source of toxic contaminants, including PAHs^8^. In China, most wastewater irrigated areas are in North China due to limited water supply. Numerous agricultural lands are irrigated with wastewater from both treated/untreated industrial or municipal effluents discharged from large sewage treatment plants^9–11^. In agricultural soils with wastewater irrigation, significant PAHs contamination is found as 0.14–2.37 mg/kg in Hunpu region and 0.18–21.02 mg/kg in Tianjin City^11^. As food chain is the most important pathways for pollutants entry and counts for 90% ingestion of contaminants into human body^12^, the uptake of carcinogenic PAHs and heavy metals through soil-to-root system and their translocation/accumulation in plant tissues has attracted many attentions, particularly for food crops cultivated on the wastewater-irrigated soils.

Persistent organic pollutants (POPs) are of high environmental and human health concerns. They are toxic and persistent in the environment, susceptible to long-range atmospheric transport, and able to bioaccumulate through food chain^13–15^. PAHs is a large class of POPs containing two or more fused aromatic rings, and 16 of them have been identified as “priority pollutants” by the United States Environmental Protection Agency (USEPA). PAHs are primarily formed and released during fossil fuels burning (coal and petroleum), waste incineration, wood processing, vehicular emissions, domestic heating, landfill and forest fires^16^. The uptake of PAHs by plants is important when considering their transfer from soils into the food chain. To protect human and animals health when exposure to PAHs contaminated soils, it is essential to understand the behaviour and restriction factors of plant uptaking PAHs^4,17^. The two well-known pathways for PAHs entrance into plants include soil-to-plant (root uptake and translocation) and air-to-plant (atmospheric deposition through stomata)^18–20^. They are the function of the physicochemical properties of PAHs such as octanol-water partition coefficient (K_ow_), environmental conditions, and plant species^21–23^. Most of hydrophilic compounds with log K_ow_ < 4 can be accumulated by roots and acropetally translocated to other plant tissues, whereas high hydrophobic non-ionized compounds with log K_ow_ > 4 may strongly partition onto the root epidermis and are unlikely to be drawn into the inner roots and translocated within the plant^24^. For instance, the non-ionizable, polar and highly water-soluble compounds, like pyrene (log K_ow_ = 4.88), are likely to be taken up by plant root and translocated to shoot^25^.

Heavy metal contamination in wastewater irrigated soils is also a widespread and serious problem in many countries^26–28^. Excessive accumulation of heavy metals in soils and plants may pose risks and hazards to humans and ecosystems through direct ingestion or contact with contaminated soils, food chain, and drinking of contaminated groundwater^29^. Plant uptake of metals is mainly dependent on the metal mobility and availability^30^, which are generally controlled by soil adsorption and desorption characteristics^31^. The key influencing factors include pH, soil organic matter (SOM), cation exchange capacity, oxidation–reduction status and the contents of clay minerals^32,33^. Among these, pH and SOM are found to play a fundamental role in determining the concentration of heavy metals in plants^33,34^. A negative correlation between soil pH and heavy metal mobility/availability has been well documented in numerous studies. For example, the availability of Zn, Cd, Ni and Cu significantly decreases with the increasing pH^35^. Apart from soil pH, SOM also affects heavy metal availability and can increase the mobility and uptake of Cd, Ni, and Zn by plant roots^36^.

Though many previous works have studied the plant uptake of PAHs and heavy metals, only the acropetal translocation of mono-contamination has been addressed and the impacts of their co-contamination are seldom mentioned. In the real world scenario, the co-existence of heavy metals and organic pollutants such as PAHs are frequently found and can be simultaneously accumulated in soils and plants^37,38^. Since PAHs and heavy metals are both dominant contaminants in industrial wastewater, the wastewater irrigated soils are posed to co-contamination which might change the behaviour of both contaminants in soil-to-root system. Cu is reported to enhance the translocation of polybrominated diphenyl ethers in maize, attributing to the root damage caused by Cu ions^39^. However, information is very limited about acropetal translocation of a series of compounds with intermediate and high lipophilicity by plants, particularly in field-contaminated soils. It is therefore of great importance to investigate the impacts of PAHs-metal co-existence on their uptake and acropetal translocation.

We hypothesize that the co-existence of PAHs and heavy metals in wastewater irrigated soils will significantly change their fate in environment and uptake by plant, comparing with the case of mono-contamination. Maize was therefore selected in this study as the model plant and collected from Chinese farmland with wastewater irrigation. By analyzing the concentration of 16 priority PAHs and 5 heavy metals in soils and maize tissues, we for the first time proved that the heavy metal Pb does significantly reduce PAHs acropetal translocation in maize, but not in reverse. Our work suggests the possible overestimation of PAHs accumulation in food crops when heavy metals co-exist and explains the potential mechanisms, showing valuable insight into wastewater irrigation management.

## Materials and Methods

### Sampling and pre-treatment

The wastewater irrigated soils were collected in September 2012 from farmlands in Tongliao city, which is located in the east of Inner Mongolia and one of the most important industrial and agricultural cities in China. The West Liao River (WLR) flows through the city and receives effluents from both treated/untreated industrial and local municipal wastewater through ditches. Due to high demand and shortage of freshwater, about 7.5 × 10^6^ m^2^ farmland is irrigated with wastewater for more than 40 years. The annual quantity of irrigation applied to the fields is about 0.9 m^3^/m^2^ ^40^, and the typical concentrations of PAHs and heavy metals in irrigated wastewater and groundwater were sampled, analyzed and listed in Supporting Information (SI) Figure S1.

The predominant soil type in study area is chestnut soil, and the main food crop is maize. Fifteen surface soils (0–20 cm) were collected at different sites in the farmlands, using a geotome in triplicates and combined as a single sample (Table S1). At five sampling points (W01, W04, W07, G04 and G08), vertical soils were collected by steel core probes (1.5 m in length and 15 cm in diameter) at nine depths (0–20 cm, 20-30 cm, 30–40 cm, 40–50 cm, 50–60 cm, 60–70 cm, 70–80 cm, 80–90 cm and 90–100 cm). After mixing well, the stones, plant roots and other large debris objects were removed and the soils were put into aluminum box. All the samples were stored at −20 °C and transferred to the laboratory until analysis.

Fifteen samples of maize tissues were collected at maturity, in triplicates from the same sites as soils. After placed in clean aluminum foil bags, the maize tissues were taken back to the laboratory and split into leaves, roots, stems and grains. All the tissues were washed with deionized water, air dried, pulverized to pass through a 40-mesh sieve, and finally stored at −20 °C until analysis.

### PAHs extraction and analysis

The extraction and clean-up of PAHs followed the previous studies^41,42^. Briefly, all the samples were sieved through a 100 mesh. For soils, roots, stems and leaves, the 10 g of samples were mixed with anhydrous sodium sulfate (previously dried at 600 °C) and spiked with a labeled surrogated standard (naphthalene-D8, fluorene-D10, pyrene-D10 and perylene-D12). All the samples were then Soxhlet extracted for 24 h with a 200 mL mixture of solvent (hexane/acetone, 1:1, v/v). The extracts were concentrated, cleaned-up and passed through a column containing 10 g of activated silica gel (Silica 60; Merck, Germany) topped with 2 g of anhydrous sodium sulfate. The extract was eluted with a 60 mL mixture of dichloromethane and hexane (1:1, v/v). The elution was finally concentrated to 1 mL under nitrogen evaporation prior to gas chromatography–mass spectrometry (GC-MS) analysis. For maize grains, 10 g samples were directly Soxhlet extracted with 200 mL mixed solvent for 24 hours, and concentrated to 3–4 mL. Clean-up was performed with gel permeation chromatography (GPC) filled with Bio-Beads s-x3. The elution was finally concentrated to 1 mL.

The content of 16 priority PAHs was analyzed by GC-MS (Agilent, 6890 N GC, 5975 MS) equipped with DB-5MS capillary column (30 m × 0.25 mm × 0.25 μm, Agilent). The carrier gas was helium (high purity, 99.999%). GC temperature was programmed as: an initial 90 °C hold for 5 min; raise from 90 °C to 180 °C by 10 °C/min and hold for 1 min; raise from 180 to 280 °C by 5 °C/min and hold for 10 min. Two ions were monitored for identifying and quantifying each PAHs. Quantification was performed by the internal standard method using a five-point calibration curve (r^2^ > 0.999). Strict quality control procedures were conducted in this study. For every set of 10 samples, a procedural blank was run to check procedural performance and matrix effects. The surrogate standard recoveries in samples ranged from 71–117%. All the PAHs concentrations reported here were recovery corrected.

### Heavy metal extraction and analysis

About 0.50 g of dried soils and tissues were ground and transferred into clean and dry digestion tubes. After added with concentrated HNO_3_ (8 mL) and stabilized overnight, the tubes were then placed on a heating block (Mars-Xpress, CEM) for digestion. The digestion temperature program was: raise from room temperature to 120 °C in 5 min and hold for 1 min; raise to 160 °C by 8 °C/min and hold for 5 min; raise from 160 °C to 185 °C by 5 °C/min and hold for 15 min. After digestion, the solutions were cooled, diluted to 50 mL with ultrapure water, and finally filtered into plastic bottles pre-washed with acid. The concentrations of heavy metals in the acid digests were measured by inductively coupled plasma mass spectrometry (ICP-MS, Agilent 7500a). For quality control, analytical blank and standard reference soil (from the Chinese National Standard Soil Bank, GBW07402 GSS-2) was performed in each sample batch to validate the digestion procedure and metal analysis. Recoveries were 83.1–109.6% for Cr, 86.2–117.4% for Ni, 84.4–109.5% for Cu, 88.1-116.4% for Pb and 91.4-108.2% for Zn. The results showed that the detected values met with the accuracy requirements comparing to the certified values. Soil pH was measured (soil:water = 1:2.5) using a pH-meter. Soil organic matter (SOM) was analyzed by the Walkley-Black method^43^ and calculated as mg per gram dry soil weight (mg/g dw).

### Data analysis

PAHs and heavy metals concentrations in the soils and maize tissues were calculated on their dry weight (dw). The bioconcentration factors of roots (RCFs), stems (SCFs), leaves (LCFs) and grains (GCFs) are used for determining the acropetal translocation of contaminants in plants, as described in Equation (1) to Equation (4):

1 $${\rm{RCFs}}=\frac{{{\rm{C}}}_{{\rm{roots}}}}{{{\rm{C}}}_{{\rm{soils}}}}$$

2 $${\rm{SCFs}}=\frac{{{\rm{C}}}_{{\rm{stems}}}}{{{\rm{C}}}_{{\rm{soils}}}}$$

3 $${\rm{LCFs}}=\frac{{{\rm{C}}}_{{\rm{leaves}}}}{{{\rm{C}}}_{{\rm{soils}}}}$$

4 $${\rm{GCFs}}=\frac{{{\rm{C}}}_{{\rm{grains}}}}{{{\rm{C}}}_{{\rm{soils}}}}$$

where C_soils,_ ${{\rm{C}}}_{{\rm{roots}}}$, ${{\rm{C}}}_{{\rm{stems}}}$, ${{\rm{C}}}_{{\rm{leaves}}}$ and ${{\rm{C}}}_{{\rm{grains}}}$ represent the PAHs and heavy metals concentrations in soils, roots, leaves, stems and grains, respectively.

### Statistical analysis

PAHs and heavy metals concentrations were standardized through logarithmic transformation by Excel 2013. Analysis of variance (ANOVA, p < 0.05) was used to evaluate the differences in the concentration of heavy metals and PAHs between wastewater- and groundwater-irrigated soils. The independent samples t-test was carried out to test the Pearson correlation between soil depth and PAHs or heavy metals, at 95% confidence level (p < 0.05) using SPSS 19.0 software. A redundancy analysis (RDA) with Monte Carlo was performed to determine the multivariate relationship between the uptake of PAHs by maize and the environmental variables (pH, SOM, PAHs, RCFs-Cr, RCFs-Ni, RCFs-Cu, RCFs-Zn and RCFs-Pb) using Canoco for Windows 4.5. The canonical axes were evaluated by “forward testing” for the significance of the successive canonical eigenvalues and environmental variables.

## Results and Discussion

### PAHs and heavy metal contamination in surface soils

The physicochemical characteristics of all the soil samples were shown in Table S2 and Figure S2(A). There was no significant difference in all the physicochemical properties between wastewater and groundwater irrigated soils. The pH value ranged from 8.09 to 8.90, indicating that the soils were alkaline in the study area. The results were similar with previous report that irrigation with treated wastewater raised the soil pH from 7.1 to 8.6^44^. The SOM was 15.58 mg/g and 20.03 mg/g for wastewater and groundwater irrigated soils, respectively. They were comparable to those in Hunpu wastewater irrigation area (12.3–31.4 mg/g)^45^, but lower than others in Beijing (70.6–80.8 mg/g)^46^ and Tianjin (averagely 26.6 mg/g)^47^.

The concentrations of all the 16 US EPA priority PAHs (total PAHs) in surface soils were listed in Table S2 (SI). In general, the concentration of total PAHs in wastewater irrigated soils ranged from 103.28 to 479.32 ng/g, whereas they varied from 140.46 to 418.32 ng/g in groundwater irrigated soils. Among them, the four most dominant PAHs were NaP (110.20 ± 26.75 ng/g), Phe (30.69 ± 4.91 ng/g), Flu (14.62 ± 2.76 ng/g) and BbF (25.10 ± 1.24 ng/g). Our results suggested that the wastewater-irrigated soils were moderately contaminated, similar with total PAHs contamination in the agricultural surface soils in Beijing (5.1–297.0 ng/g)^48^ and Tunisian (120.01–365.18 ng/g)^49^, lower than those in Tianjin (1088 ng/g)^50^ and Shenyang (950–2790 ng/g)^45^, and higher than that in Tibet (6.09 ng/g)^51^.

The composition of total PAHs (grouped by the ring number) can provide detailed information of their origins, either from petrogenic or pyrolytic sources. To better understand the source of PAHs in wastewater irrigated soils, we analyzed the PAHs composition in the irrigated wastewater and groundwater, as illustrated in Figure S1. There was no significant difference in total PAHs concentrations between wastewater and groundwater, explaining the similar profiles of congener between wastewater and groundwater irrigated soils. In the present study, the high PAHs concentrations in surface soils were derived from wastewater, similar as previous studies reported by Jin^6^ and Wang^52^. As for PAHs composition, the 2-ring PAHs were the most predominant congener, accounting for 45.24% and 31.88% of the total PAHs in wastewater and groundwater irrigated soils, respectively. The percentage of 3–4 ring PAHs were 32.29% and 41.18% for two types of soils, respectively. These PAHs (2–4 ring) were also dominant PAHs congeners in the irrigated wastewater and groundwater (Figure S1). Although the PAHs levels in this study were at medium level, the percentage of 5–6 ring PAHs in wastewater and groundwater irrigated soils (22.47% and 26.94% of total PAHs, respectively) was higher in some soils, possessing more mutagenic and carcinogenic effects^53^. In comparison with soils with wastewater irrigation, our results were similar with the surface agricultural soils in Beijing where the predominant PAHs had 2 rings (59%)^6^, but different from those in Shenyang^45^ and Tunisian^49^ where 4-ring PAHs were dominant and accounted for 36.95% and 74.83% of the total PAHs, respectively.

Across all the surface soils, a wide range of heavy metals concentrations were listed in Table S2 (SI). Of all the heavy metals, only Cr was significantly higher in wastewater irrigated soils (*p* < 0.05) than groundwater irrigated ones, explained by the higher Cr concentration in wastewater (Figure S1). The average concentrations of Ni, Cu, Zn and Pb in wastewater-irrigated soils were similar to those in groundwater-irrigated soils. Our results were similar to those in Delhi (India)^54^, but lower than the wastewater irrigated soils in Beijing^46^ and Shenyang (China)^7^. Compared to the Environmental Quality Standards set by the State Environmental Protection Administration (SEPA, 1995) for soils in China, the soils in study area were slightly contaminated by heavy metals and their concentrations were below the standard.

### Vertical distribution of PAHs and heavy metals in soils

**Figure 1:**
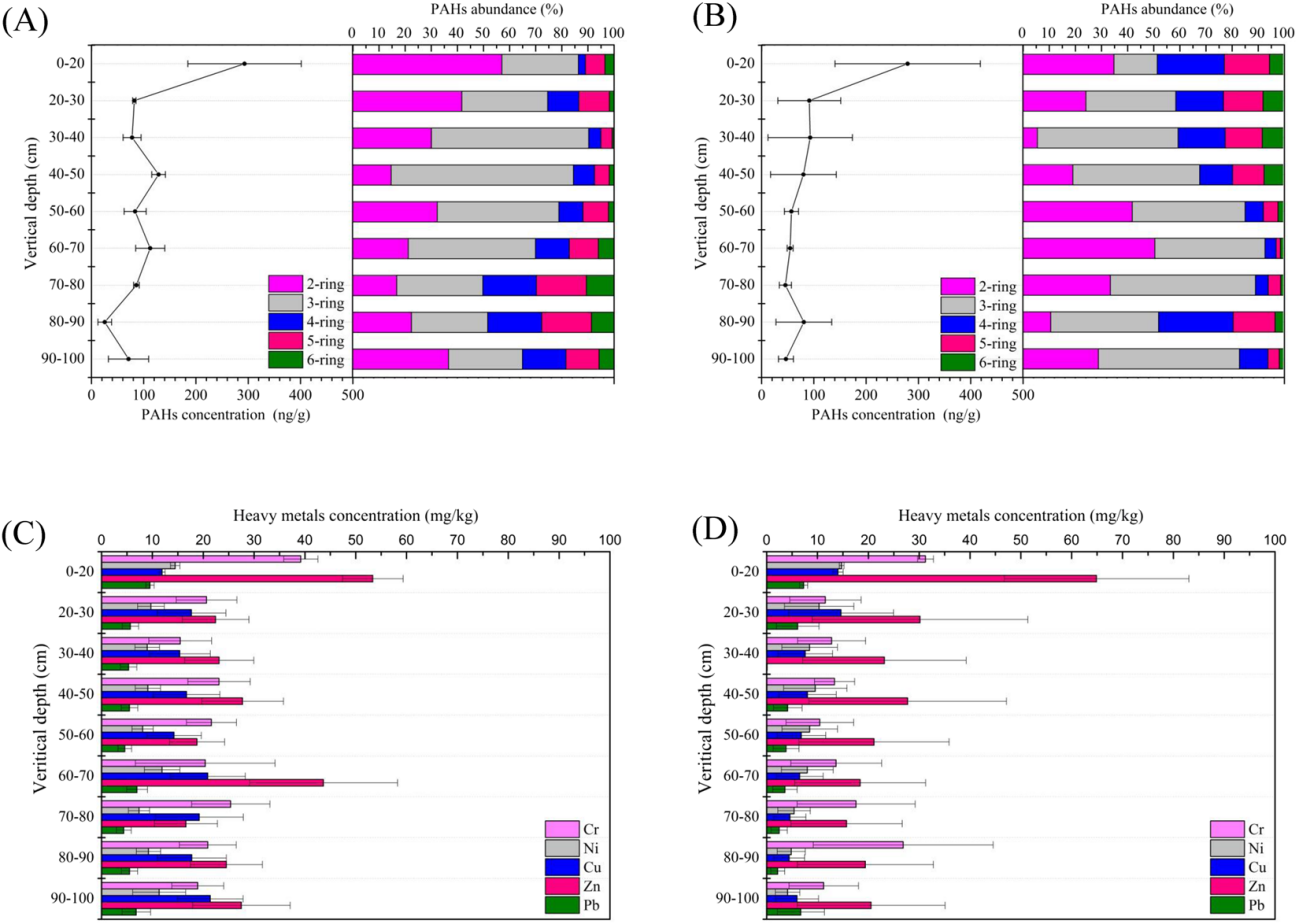
Vertical distribution of PAHs in soils irrigated with wastewater (**A**) and groundwater (**B**). In wastewater irrigated soils, PAHs correlated with soil depth include: 4-ring (Pearson correlation coefficient [PCC] = −0.823, *p* = 0.004), 5-ring (PCC = −0.658, *p* = 0.046) and 6-ring (PCC = −0.727, *p* = 0.021); in groundwater irrigated soils, PAHs correlated with soil depth include: 5-ring (PCC = −0.592, *p* = 0.084) and 6-ring (PCC = −0.867, *p* = 0.001). Vertical distribution of heavy metals in soils irrigated with wastewater (**C**) and groundwater (**D**). In wastewater irrigated soils, heavy metals correlated with soil depth include: Cu (PCC = 0.754, *p* = 0.019); in groundwater irrigated soils, heavy metals correlated with soil depth include: Ni (PCC = −0.945, *p* < 0.001), Cu (PCC = −0.862, *p* = 0.003) and Zn (PCC = −0.745, *p* = 0.021). Details see Table S3.

Figure 1(A) and (B) illustrated the vertical distribution of PAHs in wastewater and groundwater irrigated soils. In both soils, the concentrations of PAHs were highest in surface soil (0–20 cm) and declined with soil depth, following a negative correlation (*p* < 0.05). Our findings were similar with some previous studies that more PAHs in surface soils are attributed to the frequent human activities (such as irrigation) and their migration depends on the properties of both PAHs and soils^55^. Here, the percentage of 2–3 ring PAHs (log K_ow_ < 4.5) decreased with the depth, and accounted for 72.37% and 71.24% of total PAHs in wastewater- and groundwater-irrigated soils, respectively. It was worth mentioning that the soil depth had a significant negative correlation with PAHs of 4-ring (*p* = 0.004), 5-ring (*p* = 0.046) and 6-ring (*p* = 0.021) in wastewater-irrigated soils and 6-ring (*p* = 0.001) in groundwater-irrigated soils. The migration of PAHs in soils is driven by vertical water transportation and affected by SOM. SOM is reported with large sorption capacities for metals and persistent organic pollutants^56,57^. For PAHs of lower ring number (2–3 rings), they have lower log K_ow_ from 3.3 to 4.5, harder to be adsorbed by SOM and easier to migrate downward deeper soils, comparing to PAHs with higher ring number (4–5 rings) and log K_ow_ (4.9–7.1). Some previous work showed that the high SOM in Chinese farmland slows the PAHs migration comparing to the soils from urban areas^58^. Zeng *et al*. also documented that soil organic carbon is probably an important parameter influencing the vertical transport of short chain chlorinated paraffins that higher contents of soil organic carbon cause the slower migration of short chain chlorinated paraffins to deeper soils^9^. Our work is the first report demonstrating that the vertical distribution of PAHs is affected by SOM in farmland irrigated with wastewater.

Comparing to the target values of PAHs in unpolluted soils as 20–50 ng/g suggested by the Dutch Authority^59^, the PAHs concentrations of groundwater-irrigated soils deeper than 80 cm in the present study were lower, but those in wastewater-irrigated soils (70.96 ng/g) were much higher. It suggested that wastewater irrigation elevated more PAHs in deeper soils than groundwater irrigation, possessing higher risk to ecosystem and consistent with previous reports^47,60^.

As shown in Fig. 1(C) and (D), the vertical distribution of heavy metals also indicated the maximum heavy metals in surface soils (0–20 cm), except for Cu. All the other four heavy metal concentrations (Cr, Ni, Zn and Pb) slightly decreased at 30 cm and then remained stable in deeper soils. Pearson correlation analysis suggested negative correlation of Ni (*p* < 0.001), Cu (*p* = 0.003) and Zn (*p* = 0.021) in groundwater irrigated soils. No significant correlation was observed between soil depth and other heavy metals. Such surface enrichment of heavy metals might be explained by the use of fertilizer and pesticide and wastewater irrigation^61,62^. Different result was found in Shenyang, where Cu, Zn and Pb had a higher concentration in deeper soils attributing to the long pollution history of Shenyang Metal Smeltery^63^. The vertical distribution of Cu in wastewater irrigated soils was different from other heavy metals, showing higher concentration in 60–100 cm and positive correlation with soil depth (*p* = 0.019). In might be attributed to the long history of wastewater irrigation and relatively higher Cu concentration in wastewater than groundwater (Figure S1). From Kien’s study^64^, the distinct vertical distribution patterns between heavy metals might be explained by their different water-extractable fractions in soils, and metals with higher availability can migrate into deeper soils and be positively correlated with soil depth.

### Profiles of PAHs and heavy metal in maize tissues

**Figure 2:**
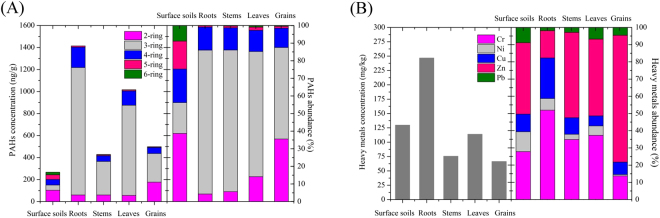
Concentration and composition of PAHs (**A**) and heavy metals (**B**) in soils and maize tissues.

There was no significant difference between maize tissues from the sites irrigated with wastewater and groundwater, and the concentrations and composition of sixteen PAHs and five heavy metals in maize tissues (roots, stems, leaves and grains) were illustrated in Fig. 2(A). Almost all the PAHs were detected in maize tissues, except for IcdP and BghiP. PAHs with 3 rings were dominant in all the maize tissues (71.40% of total PAHs), followed by 2-ring (14.86%), 4-ring (12.22%), 5-ring (1.15%) and 6-ring (0.36%). Our findings indicated that PAHs with higher ring number and K_ow_ are more difficult to be absorbed and translocated by maize tissues due to their higher hydrophobicity^65^. The higher PAHs concentrations were observed in maize roots (1414.81 ng/g) and leaves (1016.85 ng/g), because the two main pathways of organic pollutants entrance into plants include: (1) root uptake from contaminated soils: (2) deposition on leaves and uptake through stomata from the atmosphere^66,67^. The results were consistent with previous work that the highest concentration of PAHs was found in wheat roots (700.00 ng/g)^21^ or *Phragmites* roots (79.1 ng/g) and leaves (170.1 ng/g)^68^. Lower molecular weight PAHs also dominated in roots and leaves due to their high solubility and easy translocation^21^. Particularly for grains, the concentration of total PAHs was 336.29 ng/g and Phe was predominant (54.65%). Compared to previous studies, the concentration of PAHs in maize grains was comparable to that in fruit vegetables (226 ng/g, Phe 28.14%)^69^, but higher than that in wheat grains (81.2 to 95.2 ng/g, Phe 36.65%)^21^. The results illustrated the significant enrichment and accumulation of PAHs in maize grains, showing potential risks in food chain, similar as previous study^70^.

The heavy metal concentrations in maize tissues showed different trends, as illustrated in Fig. 2(B). Cr and Ni in maize tissues ranked as: roots > leaves > stems > grains, and their concentrations in the tissues were 76.34–391.69 mg/kg and 6.21–71.06 mg/kg, respectively. The highest Pb concentration was found in leaves (7.71 mg/kg), probably attributed to automotive exhausts^71^. As the essential micronutrients for plant^72^, Zn and Cu were more abundant in grains, ranging from 37.69 to 68.85 mg/kg and 3.83 to 6.14 mg/kg, respectively. The concentrations of all the detected heavy metals in the present study were comparable to the previous studies in maize^73^ and wheat grains^74^, but much lower than Asgari’s work on wheat except for Zn^75^. Particularly, the concentrations of Cr and Pb in grains ranged from 3.78 to 20.75 mg/kg and 1.30 to 5.15 mg/kg, respectively, much higher than the maximum permitted levels of China and European Union in the mature stage (SI, Table S4), showing significant accumulation in food chain and possessing potential risks to human health.

### Acropetal translocation of heavy metals and PAHs in maize

The different profiles of PAHs and heavy metals in soils and maize issues indicated their selective acropetal translocation. Such translocation is not only dependent on their contamination and type, but also the plant species. Since there was no previous report on PAHs acropetal translocation in maize, we for the first time obtained these data and compared with other crops and vegetables. The RCFs of Phe and Flu were about 10 times and 4 times higher than those in wheat, whereas the RCFs of Nap, Ace, Ant, Pyr, B(a)t and D(ah)a were comparable and the RCFs of Flu, Chr, BbF, BkF, BaP and BghiP were lower^28^. Comparing to another work on PAHs uptake by wheat^21^, our RCFs of Nap, Acy, Phe, Ant, Flu and Pyr were of similar level, but they were much lower for Ace, Flo, B(a)t and Chy. The RCFs of Ace, Flu, BaP and I(123-c,d)P for potato ranged from 0.001 to 0.1^76^ and the RCFs of Acy, Ace, Flo, Phe, Ant, Pyr, B(a)t and Chr for wetland plants ranged from 0.2 to 9.3^68^, which were all much lower than our present study. Such different behaviour might be explained by the discriminated root lipids among plants species. Gao *et al*. compared the PAHs uptake by various plant species in the same soils, suggesting that RCFs of Phe and Pyr varied significantly among these plants^77^.

**Figure 3:**
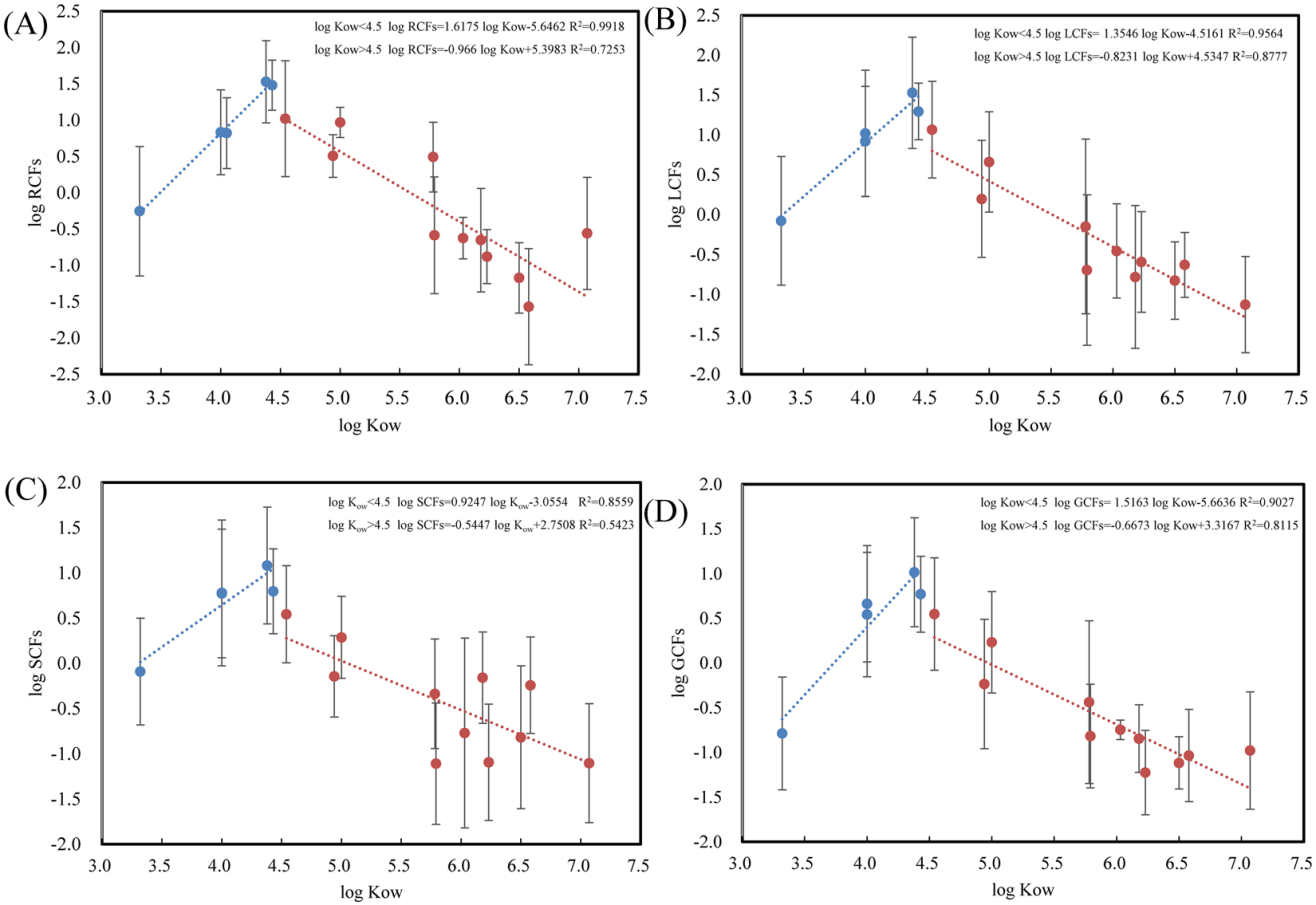
Relationship between log K_ow_ and log RCFs (**A**), log LCFs (**B**), log SCFs (**C**) and log GCFs (**D**) of PAHs in maize tissues.

The relationship between BCFs (log RCFs, log SCFs, log LCFs and log GCFs) and log K_ow_ of each PAHs was illustrated in Fig. 3. Among the 16 PAHs, Phe (log K_ow_ = 4.43) showed the maximum bioconcentration factors of 1.48 (log RCFs), 0.80 (log SCFs), 1.29 (log LCFs) and 0.77 (log GCFs). Generally, the 3-ring PAHs (Acy, Ace, Flo, Phe and Ant) with medium log K_ow_ (4.00–4.54) showed relatively higher RCFs, SCFs, LCFs and GCFs than those of the 2-ring PAHs (NaP) with lower log K_ow_ (3.32) or 5–6 ring PAHs (BbF, BkF, BaP, DahA, IcdP and BghiP) with higher log K_ow_ (6.03 to 7.07). A positive correlation between log K_ow_ and log RCFs (A), log LCFs (B), log SCFs (C) and log GCFs (D) of PAHs in maize tissues (Fig. 3) was observed when log K_ow_ is less than 4.5 (n = 15, R^2^ = 0.916–0.999, *p* < 0.05). For log K_ow_ above 4.5, they oppositely behaved the negative relationship (n = 15, R^2^ = 0.646–0.952, *p* < 0.05). Our results suggested that 3-ring PAHs with log K_ow_ around 4.5 are more likely to be uptaken by maize than 2-, 4-, 5- or 6-ring PAHs with lower or higher log K_ow_. More hydrophilic organic chemicals (log K_ow_ < 3.5) are reported to have high potential for root uptake and translocation. Some research suggested the negative correlation between log K_ow_ and log RCF when log K_ow_ is above 4.5^78^. The higher molecular weight PAHs have higher hydrophobic properties, resulting in the difficulties in translocation in vegetables^65^. However, some more complicated relationships were found between K_ow_ and BCFs recently. Lin *et al*. suggested a positive linear relationship between log RCFs and log K_ow_ of Nap, Ace, Flu, Phe and Pyr by hydroponically cultivated tea plant^79^. Although there was no linear relationship between log RCFs and log K_ow_ in the present study, we observed a bell-shape of PAHs bioconcentration factors against log K_ow_ peaking at log K_ow_ = 4.0–4.5. Tao *et al*. also identified a bell-shape relationship between log RCFs and log K_ow_ of 14 PAHs in wheat, but peaked at log K_ow_ = 5.0–5.5^80^. The log RCFs of 3-ring PAHs (Ace, Flu, Phe and B(a)t) for wheat were in the range of 0.6–0.8 and comparable to the present study, but the RCFs of 2- and 4–6 ring PAHs were much higher^80^. Intensive studies have been conducted to reveal the pathways of chemical compounds reaching aerial plant organs^17,78^. From the correlation between BCFs in Table S5 (SI), log RCFs, log SCFs, log LCFs and log GCFs have significantly positive correlation with each other (*p* < 0.01), hinting that soil-root uptake and soil volatilization is the main route for PAHs translocation into leaves, stems and grains.

**Figure 4:**
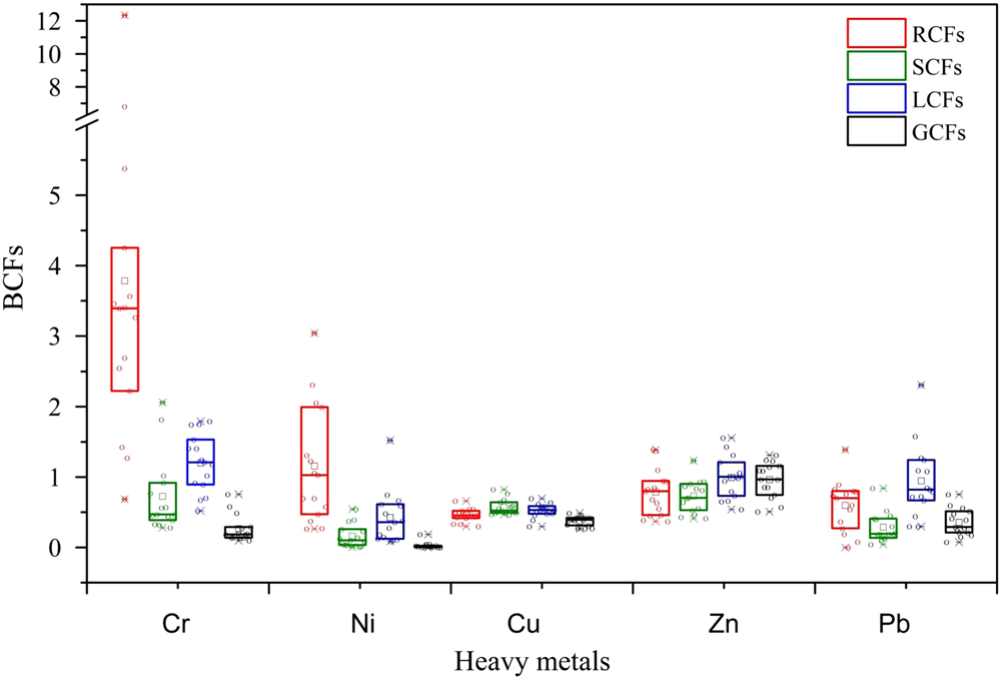
RCFs, SCFs, LCFs and GCFs of five heavy metals (Cr, Ni, Cu, Zn and Pb) in maize tissues.

The BCFs of heavy metals also represent their unique uptake behaviour by maize (Fig. 4). Cu, Zn and Pb exhibited the lowest RCFs (0.45, 0.78 and 0.60, respectively), whereas the RCFs of Ni (1.16) and Cr (3.78) were above 1.0. The BCFs of Cr and Ni followed the order: RCFs > SCFs > LCFs > GCFs, whereas they were not significantly different for Cu, Zn and Pb. Thus, the heavy metals bioaccumulation followed the order: Zn > Cr > Cu > Pb > Ni (SCFs) and Zn > Cu > Pb > Cr > Ni (GCFs). The huge difference in BCFs in the present study indicated a metal-dependent absorption capacity, which has been proved by previous work^81,82^. Meanwhile, the BCFs of heavy metals are reported to vary among crop species^46,83^. Zn and Cu, as essential elements to maize, had the highest GCFs in maize (0.38 and 0.96, respectively), comparable to the uptake by wheat (0.35 and 0.53, respectively)^84^. Here, the GCFs of Ni (0.03) were lower than the reported values in maize (0.46–0.64)^46^ but higher than those in wheat (0.004)^85^. The GCFs of Cr ranged from 0.10 to 0.76, similar as the reported values in maize (0.24)^46^ and higher than those in wheat (0.002)^85^. The GCFs of Pb (0.35) were comparable to those in vegetables (0.21 to 0.35)^86^, higher than those in wheat (0.007)^85^ and maize (0.09)^46^, and lower than those of carrot^76,87^.

### Impacts of heavy metals on PAHs acropetal translocation

**Figure 5:**
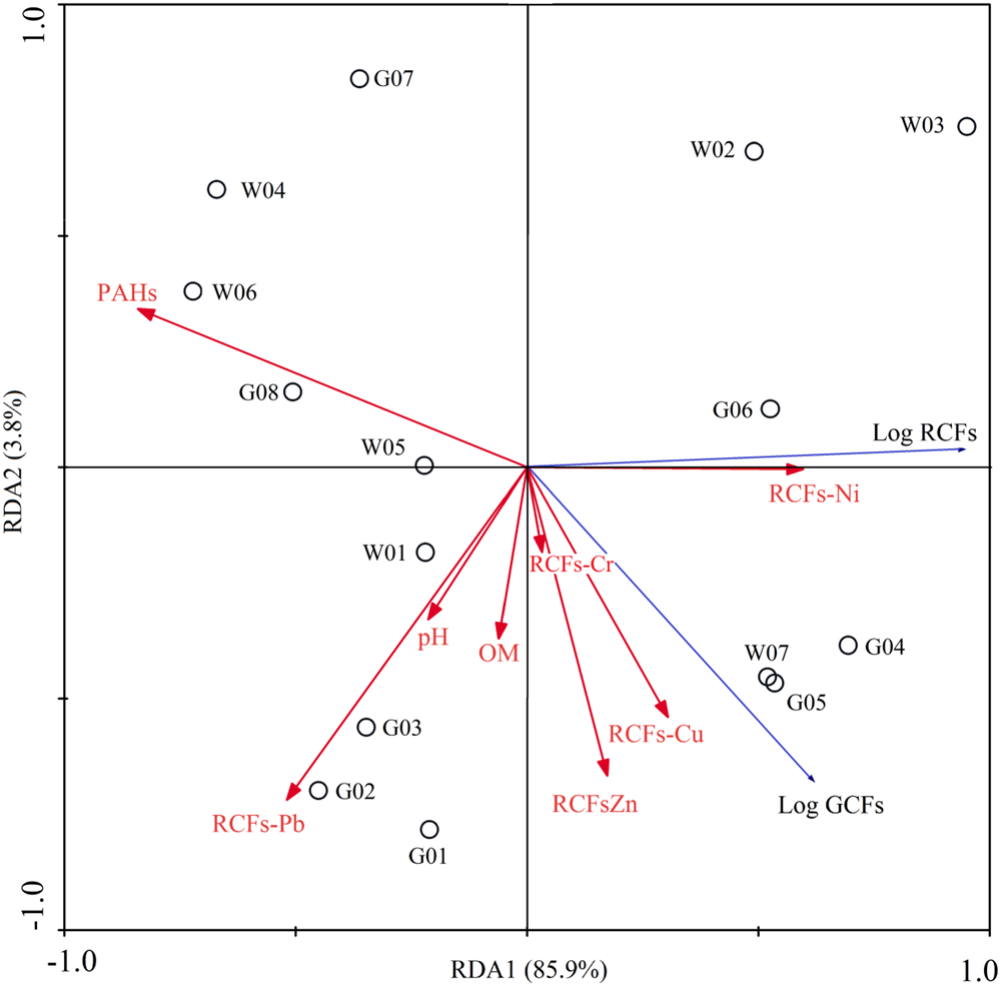
Redundancy analysis (RDA) triplot summarizing variation in PAHs uptake by maize across the soil properties and heavy metal uptake. PAHs and RCFs-Pb are included as explanatory variables that account for 61% (*p* < 0.01) and 20% (*p* < 0.01) of the total variance. Circles represent soils at different sites; red and blue arrows illustrate maize uptake of PAHs and heavy metals, respectively. Eigenvalues for the first two environmental gradients are presented.

Considering the co-contamination of PAHs and heavy metals in the wastewater-irrigated soils, further analysis was carried out to distinguish the impacts of soil properties and contaminants on PAHs uptake by maize. Since detrended correspondence analysis (DCA) showed that the lengths of first ordination gradient were less than 3, RDA was adopted to examine the correlations between the PAHs uptake by maize and environmental variables (e.g. SOM, pH, PAHs and BCFs of metals, as listed in SI Table S6). The first and second axis of RDA plot (Fig. 5) accounted for 85.9% and 3.9% of the total variance, respectively. Vectors of RCFs-Ni, RCFs-Cu and RCFs-Zn pointing in similar direction (acute angle less than 90°) with log GCFs and log RCFs of PAHs indicated their high positive relationship. On the contrast, vectors of total PAHs, RCFs-Pb and pH pointed an opposite direction, illustrating their negative correlation with log GCFs and log RCFs of PAHs.

SOM and pH are reported as the most important factors on plant uptake of PAHs and heavy metals. Hydrophobic organic compounds in soils tend to be adsorbed by SOM, and their uptake by plant roots is essentially determined by SOM rather than their concentrations^88,89^. The influence of soil pH on PAHs is based on absorption kinetics^90^, which is considered as one of the most important factors affecting the uptake of pollutants by plants. It is well known that the plant uptake and translocation for weak ionizable organic compounds decrease with the increasing pH, attributing to the ion-trap mechanism speculating that the anions across the root cell membranes is weakened by passive diffusion of the undissociated acids^91,92^. The mobility and bioavailability of heavy metals also increase with decreased soil pH^93–95^, which affects the uptake of heavy metals by plants^96,97^ via altering metal speciation, solubility from mineral surfaces, and eventual bioavailability^98,99^. Different from previous research, neither SOM nor pH showed significant impacts on PAHs uptake in the present study, only explaining 2.74% and 1.39% of variance in RDA (Table S6). Instead, among all the environmental variables selected, the two significant ones (*p* < 0.01) explaining the majority of variance were PAHs (61%) and RCFs-Pb (20%). The less impacts of pH might be explained by the small variation of soil pH, ranging from 8.085 to 8.895. According to previous studies, the RCFs of perfluorooctane sulfonate by maize remained unchanged when pH ranged from 6 to 8, only significantly changing at lower or higher pH values^92^. The negative effects of RCFs-Pb indicated that PAHs-metal co-contamination reduced the PAHs uptake. One possible reason is the competitive interaction of heavy metals and PAHs. The adsorption (both exchange and specific ones) of PAHs on soil aggregates is determined by the amounts, structures and availability of active binding sites^100^. Metal ion binding to SOM can change functional groups and surface complexation^99^, further altering their interaction with PAHs. Meanwhile, the electrostatic interaction or formation of hydrogen bond on soil clays also contributes to PAHs adsorption^101^, which is influenced by heavy metals. Another possible mechanism is that heavy metals might damage root cell membrane in defective root system, which significantly enhances the uptake of polybrominated diphenyl ethers (PBDEs) by maize^39^. For the first time, our work reports the similar behaviour in maize cultivated in farmlands irrigated with wastewater, and the findings further suggest that the impacts of heavy metals on PAHs uptake are negligible and might even be the main driven force in agricultural soils with PAHs-metal co-contamination or irrigated with wastewater.

## Conclusions

The present study evaluates the co-occurrence of PAHs and heavy metals in soils irrigated with wastewater in Inner Mongolia and studied their uptake and translocation by maize. We find that PAHs and heavy metals in surface soils showed moderately contamination, and their accumulation in maize grain posed health risks. Logarithm bioconcentration factors were positively correlated with log K_ow_ when it is less than 4.5, while the negative correlation was found for log K_ow_ > 4.5. RDA results illustrated limited impacts of pH and SOM on PAHs uptake by maize, although they are identified as the key environmental variables affecting PAHs uptake in mono-PAHs contamination. Instead, the Pb uptake was identified as one of the most significant factors, hinting that PAHs-metal co-contamination alters PAHs uptake attributing to the metal-soil or metal-root interactions. Our findings change our understanding on PAHs uptake in PAHs-metal co-contaminated farmland with wastewater irrigation, and broaden our vision about the fate of PAHs in agricultural soils. Our work also hints the mis-estimation of PAHs uptake by crops in case of co-contamination, which might contribute to the practical agricultural management, particularly in case of wastewater irrigation.

## Electronic supplementary material

Supplementary Information
