# Supplementary material for: Uptake and translocation of polycyclic aromatic hydrocarbons (PAHs) and heavy metals by maize from soil irrigated with wastewater
Source: Sci Rep. 2017 Sep 22;7:12165. doi: 10.1038/s41598-017-12437-w (PMC5610240; doi:10.1038/s41598-017-12437-w)
Supplement: Supplementary file 1 — Supplementary Information [file 41598_2017_12437_MOESM1_ESM.doc]

**Supporting Information**

**Uptake and translocation of polycyclic aromatic hydrocarbons (PAHs) and heavy metals in maize from soil irrigated with wastewater**

Shichao Zhang^a,c^, Hong Yao^a,*^, Yintao Lu^a^, Xiaohua Yu^a,c^, Jing Wang^a^, Shaobin Sun^a^, Mingli Liu^a^, Desheng Li^a^ ,Yi-Fan Li^d^, Dayi Zhang^b,c,*^

^a^ Beijing Key Laboratory of Aqueous Typical Pollutants Control and Water Quality Safeguard, School of Civil Engineering and Architecture, Beijing Jiaotong University, Beijing 100044, PR China.

^b^ School of Environment, Tsinghua University, Beijing, 100086, PR China.

^c^ Lancaster Environment Centre, Lancaster university, Lancaster, LA1 4YQ, UK.

^d^ IJRC-PTS, School of Municipal and Environmental Engineering, Harbin Institute of Technology, Harbin 150090, China.

***Corresponding author**

Dr Hong Yao, Beijing Key Laboratory of Aqueous Typical Pollutants Control and Water Quality Safeguard, School of Civil Engineering and Architecture, Beijing Jiaotong University, Beijing 100044, PR China; Tel.: +86(0) 1051682157; Fax: +86(0)1051682157; Email: yaohongts@163.com

Dr Dayi Zhang, School of Environment, Tsinghua University, Beijing, 100086, PR China; Lancaster Environment Centre, Lancaster University, Lancaster, LA1 4YQ, UK; Tel.: +44(0)1524510288; Fax: +44(0)1524510082; Email: [d.zhang@lancaster.ac.uk](mailto:d.zhang@lancaster.ac.uk)

Pages: 9

Figures: 2

Tables: 6

**
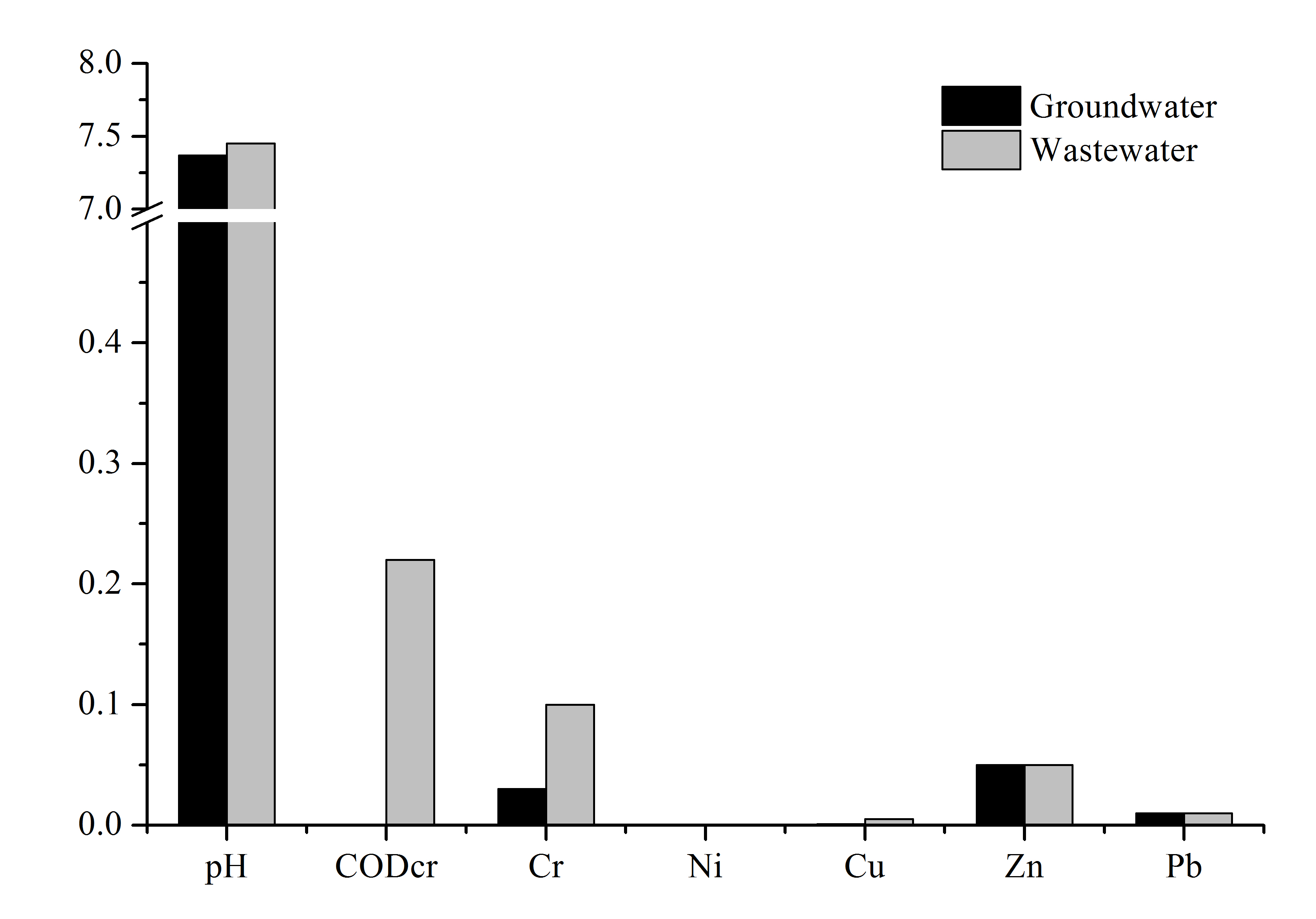
** **
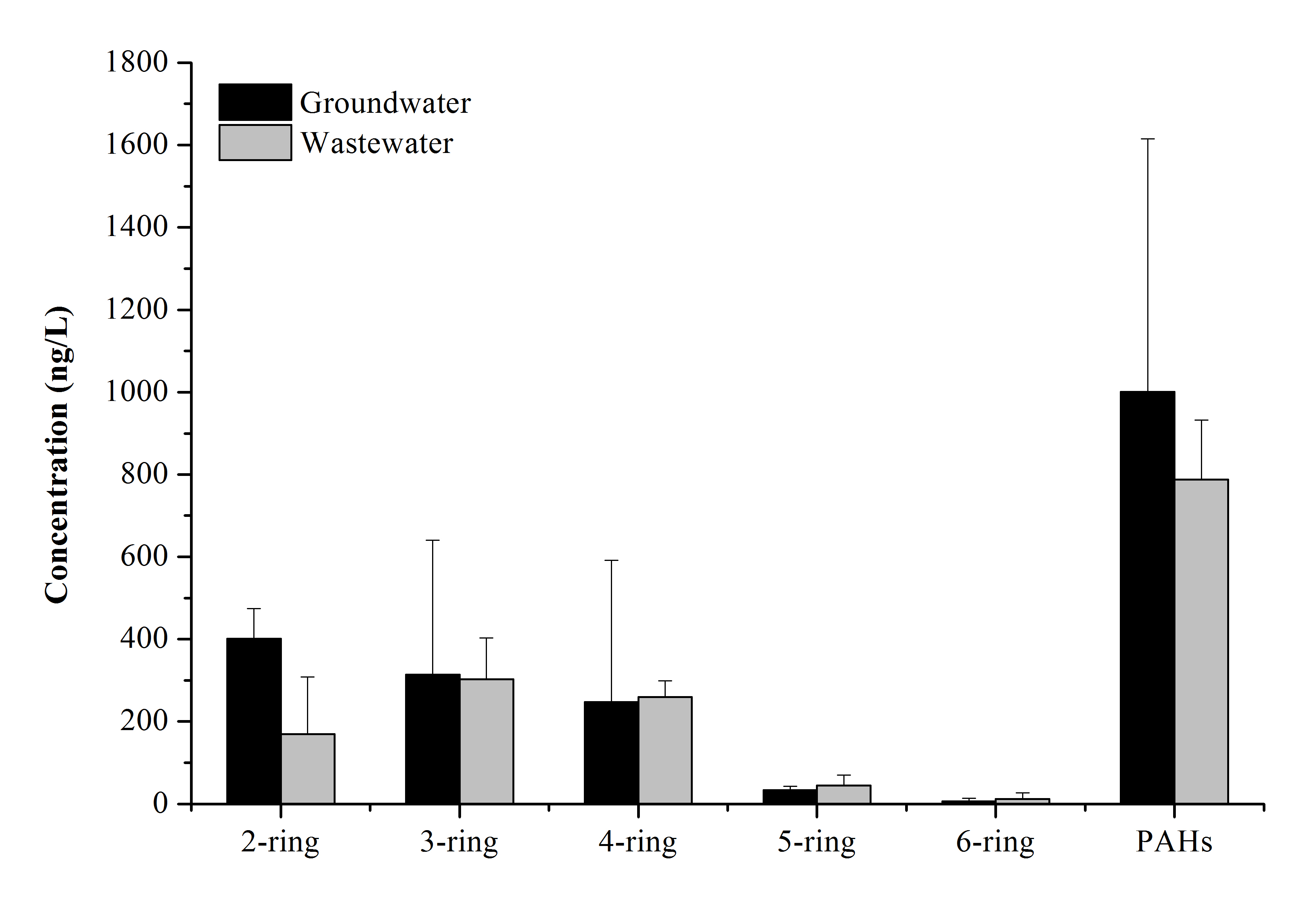
**

(A) (B)

**Figure S1.** (A) The concentrations of heavy metals (Cr, Ni, Cu, Zn and Pb) in irrigated wastewater and groundwater. (B) The concentrations of PAHs with different ring numbers in irrigated wastewater and groundwater.


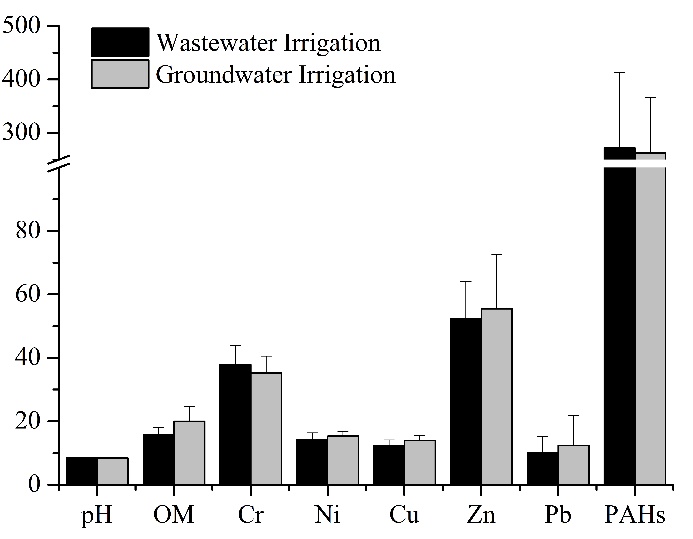

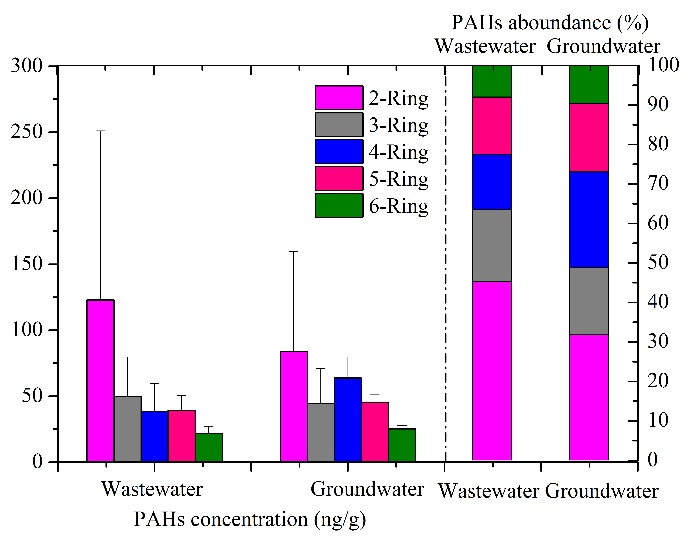


(A) (B)

**Figure S2.** (A) The concentrations of heavy metals (Cr, Ni, Cu, Zn and Pb) in surface soils with wastewater and groundwater irrigation. (B) The concentration and abundance of PAHs with different ring number in wastewater- and groundwater-irrigated soils.

**Table S1.** Sampling sites.

| **Site** | **Longitude** | **Latitude** | **Irrigation type** | **Samples** | |
| --- | --- | --- | --- | --- | --- |
|  |  |  |  | **Top** | **Vertical** |
| **W01** | 43°40.688′ | 122°24.337′ | Wastewater | Yes | Yes |
| **W02** | 43°40.709′ | 122°24.517′ | Wastewater | Yes | - |
| **W03** | 43°40.715′ | 122°24.672′ | Wastewater | Yes | - |
| **W04** | 43°41.067′ | 122°16.511′ | Wastewater | Yes | Yes |
| **W05** | 43°40.919′ | 122°16.496′ | Wastewater | Yes | - |
| **W06** | 43°40.934′ | 122°16.648′ | Wastewater | Yes | - |
| **W07** | 43°30.526′ | 122°23.503′ | Wastewater | Yes | Yes |
| **G01** | 43°40.036′ | 122°24.361′ | Groundwater | Yes | - |
| **G02** | 43°40.032′ | 122°24.391′ | Groundwater | Yes | - |
| **G03** | 43°40.061′ | 122°24.367′ | Groundwater | Yes | - |
| **G04** | 43°41.082′ | 122°16.183′ | Groundwater | Yes | Yes |
| **G05** | 43°40.017′ | 122°16.063′ | Groundwater | Yes | - |
| **G06** | 43°41.053′ | 122°59.912′ | Groundwater | Yes | - |
| **G07** | 43°30.464′ | 122°23.348′ | Groundwater | Yes | - |
| **G08** | 43°30.506′ | 122°23.072′ | Groundwater | Yes | Yes |

**Table S2.** The concentrations of total PAHs and heavy metals in top soils.

| **Site** | **Wastewater irrigation^1^** | **Groundwater irrigation^1^** | ***p*-value** |
| --- | --- | --- | --- |
| **pH** | 8.09-8.90 (8.43±0.25) | 8.18-8.56 (8.44±0.12) | 0.100 |
| **SOM (mg/g)** | 11.54-18.99 (15.58±2.39) | 14.61-29.61 (20.03±4.63) | 0.420 |
| **Cr (mg/kg)** | 27.70-46.66 (37.14±6.24) | 27.46-43.39 (35.23±5.29) | 0.045* |
| **Ni (mg/kg)** | 11.88-17.41 (14.67±1.93) | 13.64-17.76 (15.32±1.55) | 0.653 |
| **Cu (mg/kg)** | 10.36-15.19 (12.55±1.81) | 11.08-15.57 (13.96±1.64) | 0.141 |
| **Zn (mg/kg)** | 41.20-74.04 (52.18±12.64) | 39.24-90.56 (55.47±17.03) | 0.089 |
| **Pb (mg/kg)** | 5.88-21.32 (10.50±5.35) | 5.34-30.97 (12.38±9.38) | 0.160 |
| **Total PAHs (ng/g)** | 103.28-479.32 (294.03±51.41) | 140.46-418.32 (262.52±36.74) | 0.800 |

^1^ Data are presented in range (mean ± standard deviation).

* Correlation is significant at the 0.05 level (2-tailed).

**Table S3.** The correlation of PAHs and heavy metals concentrations with depth in vertical soils.

|  | | **PAHs** | | | | | **Heavy metals** | | | | |
| --- | --- | --- | --- | --- | --- | --- | --- | --- | --- | --- | --- |
|  |  | **2-ring** | **3-ring** | **4-ring** | **5-ring** | **6-ring** | **Cr** | **Ni** | **Cu** | **Zn** | **Pb** |
| **Wastewater irrigation** | PCC | 0.511 | 0.282 | -0.826^**^ | 0.661 | -0.698^*^ | -0.469 | -0.333 | 0.754^*^ | -0.389 | -0.377 |
|  | *p*-value | 0.159 | 0.462 | 0.006 | 0.053 | 0.036 | 0.202 | 0.381 | 0.019 | 0.300 | 0.317 |
| **Groundwater irrigation** | PCC | 0.059 | 0.650 | -0.344 | -0.605 | -0.732^*^ | -0.186 | -0.945^**^ | -0.862^**^ | -0.745^*^ | -0.215 |
|  | *p*-value | 0.879 | 0.058 | 0.365 | 0.084 | 0.025 | 0.631 | 0.000 | 0.003 | 0.021 | 0.579 |

PCC: Pearson correlation coefficient.

^*^ Correlation is significant at the 0.05 level (2-tailed).

^**^ Correlation is significant at the 0.01 level (2-tailed).

**Table S4.** The maximum permitted levels of heavy metal concentrations (mg/kg) in maize grain in China and the European Union.

| **Heavy metals** | **Safe limits of cereals** | |
| --- | --- | --- |
|  | **China (GB2762-2012)** | **European Union (EC: No 1881/2006)** |
| **Cr** | 1 | - |
| **Ni** | - | - |
| **Cu** | - | - |
| **Zn** | - | - |
| **Pb** | 0.2 | 0.20 |

**Table S5.** The correlation among log RCFs, log LCFs, log SCFs and log GCFs of PAHs.

|  | **log RCFs** | **log LCFs** | **log SCFs** | **log GCFs** |
| --- | --- | --- | --- | --- |
| **log RCFs** | 1.000 | - | - | - |
| ***p*-value** | - | - | - | - |
| **log LCFs** | 0.912^**^ | 1.000 | - | - |
| ***p*-value** | <0.001 | - | - | - |
| **log SCFs** | 0.811^**^ | 0.926^**^ | 1.000 | - |
| ***p*-value** | <0.001 | <0.001 | - | - |
| **log GCFs** | 0.936^**^ | 0.971^**^ | 0.917^**^ | 1.000 |
| ***p*-value** | <0.001 | <0.001 | <0.001 | - |

** Correlation is significant at the 0.01 level (2-tailed).

**Table S6.** Summary of the forward selection procedure in the redundancy analysis (RDA).

| **Environmental variable** | **E (%)^1^** | **P^2^** | **F^3^** |
| --- | --- | --- | --- |
| **PAHs** | 61 | 0.002** | 20.53 |
| **RCFs-Pb** | 20 | 0.004** | 12.20 |
| **pH** | 4 | 0.104 | 2.74 |
| **OM** | 1 | 0.280 | 1.39 |
| **RCFs-Cu** | 2 | 0.288 | 1.25 |
| **RCFs-Ni** | 1 | 0.658 | 0.32 |
| **RCFs-Zn** | 0 | 0.468 | 0.60 |
| **RCFs-Cr** | 1 | 0.814 | 0.14 |

^1^ E represents the variation explained by the selected variables.

^2^ P refers to the significance of explained variation.

^3^ F is the probability distribution of F test. The values of *p* and F were tested using Monte Carlo permutation test with 499 permutations.

** Correlation is significant at the 0.01 level (2-tailed).
